# Pan-cancer analysis identifies SPEN mutation as a predictive biomarker with the efficacy of immunotherapy

**DOI:** 10.1186/s12885-023-11235-0

**Published:** 2023-08-24

**Authors:** Ya-Dong Li, Hao Huang, Zheng-Ju Ren, Ye Yuan, Hao Wu, Chuan Liu

**Affiliations:** Department of Urology, The Second Affiliated Hospital, Chongqing Medical University, Chongqing, China

**Keywords:** SPEN, X chromosome inactivation, Immune checkpoint inhibitor, Immunotherapy, Prognosis

## Abstract

The association between specific genetic mutations and immunotherapy benefits has been widely known, while such studies in pan-cancer are still limited. SPEN, mainly involved in X chromosome inactivation (XCI), plays an essential in tumorigenesis and sex differences in cancer. Thus, we firstly analyzed the potential role of SPEN in the TCGA pan-cancer cohort and clinical samples. Bioinformatics analysis and immunohistochemistry (IHC) staining confirm that the expression of SPEN is significantly different in various cancers and may involve RNA splicing and processing via enrichment analysis. Then, our data further revealed that those patients with SPEN mutation could predict a better prognosis in pan-cancer and had distinct immune signatures, higher tumor mutation burden (TMB), and microsatellite instability (MSI) in common cancer types. Finally, the cancer patients from 9 studies treated with immune checkpoint inhibitors were included to investigate the efficacy of immunotherapy. The results further showed that SPEN mutation was associated with better clinical outcomes (HR, 0.74; 95%CI, 0.59–0.93, *P* = 0.01), and this association remained existed in female patients (HR, 0.60; 95%CI, 0.38–0.94 *P* = 0.024), but not in male patients (HR, 0.82; 95%CI, 0.62–1.08 *P* = 0.150). Our findings demonstrated that SPEN mutation might strongly predict immunotherapy efficacy in pan-cancer.

**Supplementary Information:**

The online version contains supplementary material available at 10.1186/s12885-023-11235-0.

## Introduction

The advent of immune checkpoint inhibitors (ICIs) has improved the survival of patients with advanced cancers [1]. These inhibitors, including anti-cytotoxic T-lymphocyte-associated antigen 4 (CTLA-4), anti-programmed cell death receptor-1 (PD-1), or its ligand (PD-L1) induced T cell activation mechanisms to mediate anti-tumor response, and thus has been used for the treatment in multiple cancers [2, 3]. However, only about 15%-20% of patients with advanced cancers could benefit from ICIs [4–6], and identifying predictive biomarkers is critical. To date, PD-L1 expression, tumor mutation burden (TMB), and microsatellite instability (MSI) have been used to predict the response to ICIs [7–9], but these do not necessarily preclude or not always correlate with clinical response [10, 11]. Thus, additional biomarkers contributing to ICIs response seems also become important.

SPEN, also known as SMRT/HDAC1-associated repressor protein (SHARP), is a nuclear protein of more than 400 kDa with crucial roles in X-linked gene silencing and transcriptional regulation [12–14]. SPEN contains four N-terminal RRMs (RNA recognition motifs) and a highly conserved C-terminal SPOC domain involved in the Notch signaling pathway and nuclear receptor signaling [15, 16]. The prior studies reported that SHARP is involved in nuclear receptor signaling by recruiting the corepressor SMRT complex via its SPOC domain [15]. It has been reported that SPEN was critical in regulating embryogenesis and throughout development via involvement in the Notch signaling pathway [16–18]. Recently, Feng et al. reported that SHARP is an essential positive regulator of Wnt signaling in cancers with β-catenin dysregulation [19]. Legare et al. revealed that the inactivation of SPEN by 23% deletion of heterozygosity and/or 3% to 4% somatically acquired mutations may contribute to breast tumor formation and progression [20]. In addition, SPEN mutations have also been reported in diffuse large B-cell lymphoma (DLBCL) [21], splenic marginal zone lymphoma (SMZL) [22, 23], and pancreatic carcinoma [24]. Although more and more studies demonstrated that the occurrence of SPEN mutations is very high in cancer, there have been no comprehensive pan-cancer studies on SPEN mutation. Thus, we aimed to investigate the association between SPEN mutation and the prognosis and immunotherapy in human cancer.

## Methods

### Data collection

The RNA expression and clinical data of The Cancer Genome Atlas (TCGA) and corresponding normal samples data of the Genotype-tissue expression (GTEx) database were obtained from the UCSC Xena database (https://xenabrowser.net/datapages/). All data included for prevalence analysis of SPEN mutations and copy number alterations (CAN), subtype analysis, 3D protein structure, mutation counts, MSIsensor score, MSI MANTIS score, and survival analysis were downloaded from the cBioPortal for Cancer Genomics database (https://www.cbioportal.org) [25]. To explore the association between SPEN mutation and immune characteristic, the data including STAD, BRCA, SKCM, COAD, BLCA, HNSC, and LUAD, was obtained from TCGA by using TISIDB (http://cis.hku.hk/TISIDB) [26].

### The protein expression analysis of SPEN

The protein expression analysis of SPEN was explored through the clinical proteomic tumor analysis consortium (CPTAC) dataset (http://ualcan.path.uab.edu/). The expression level of total protein of SPEN between primary tumor and normal tissues was explored. The following four cancers were included: clear cell renal cell carcinoma (ccRCC), breast cancer, ovarian cancer, and Glioblastoma multiforme (GBM).

### Immunohistochemical staining

From May 2021 to May 2022, 29 paraffin-embedded tumor specimens and 12 cases of normal tissue were collected from the Second Affiliated Hospital of Chongqing Medical University. These patients were newly diagnosed and pathologically confirmed to be cancers.

Tissues of different cancers and corresponding normal tissues were incubated with appropriately primary antibodies against SPEN (Abcam #ab72266)) at 37℃ and then 4°C overnight. After washing 3 times with PBS for 10 min, the sections were incubated with secondary antibody at 37℃ for 60 min. After washing three times with PBS for 10 min, the tissues were stained with DAB and hematoxylin. Finally, the sections were observed with microscopy. IHC staining scores were determined by the intensity of SPEN and the percentage of positive tumor cells, and the multiplication between the two was total scores of each visual field.

### Prognostic analysis

Kaplan–Meier analysis was performed to evaluate the overall survival (OS) of patients from the TCGA cohort. Univariate Cox regression analyses were conducted to assess the significance of SPEN in predicting OS, disease-specific survival (DSS), disease-free survival (DFS), and progression-free survival (PFS) in pan-cancer using the R package“survival” and “survminer”.

### Genetic alteration analysis

The cBioPortal for Cancer Genomics database (https://www.cbioportal.org) was used to analyze and download the data, including prevalence analysis of SPEN alteration, subtype analysis, 3D protein structure, TMB, MSIsensor score, MSI MANTIS score, and survival analysis.

### Gene-related enrichment analysis

The experimentally determined SPEN-binding proteins were obtained via the STRING website (https://string-db.org/) and the top 100 SPEN-correlated targeting genes and correlation analysis of SPEN and selected genes from the TCGA cohort were downloaded via the GEPIA website (http://gepia.cancer-pku.cn/). Then, the Kyoto Encyclopedia of Genes and Genomes (KEGG) pathway and Gene Ontology (GO) Enrichment analysis was conducted using the R package“clusterProfiler” [27].

### Immune cell infiltration

All data on the immune cell infiltration score of TCGA from the TIMER2 database (http:// timer.cistrome.org/) was downloaded. The relationship between the level of SPEN expression and the abundance of TIICs, including CD4 + T cells, CD8 + T cells, B cells, neutrophils, dendritic cells (DCs), and macrophages, was analyzed. The results were obtained with the “estimate” R package and presented with ImmuneScore, StromalScore, and ESTIMATEScor. Tumor Immune Dysfunction and Exclusion were analyzed by using TIDE algorithm (http://tide.dfci.harvard.edu/). The gene with high-scoring in TIDE signatures might impact resistance to cancer immunotherapy and tumor immune escape. In this study, the TIDE algorithm was used to evaluate associations between SPEN expression and immunosuppression.

### Data analysis of patients with immunotherapy

The completed clinical trials about immune checkpoint blockade across all cancer types were searched on ClinicalTrials.gov, which included anti-PD-L1 (avelumab, atezolizumab, and durvalumab), anti-PD-1 (nivolumab, pembrolizumab, and cemiplimab), and anti-CTLA-4 (ipilimumab and tremelimumab). Then, a search of PubMed for potential trials was performed from inception to June 2022. Two reviewers (YDL and HH) independently screened the full texts for potentially relevant studies. Any discrepancy was resolved by discussion. To be eligible, trials had to meet the following criteria: (1) population: clinical trials recruiting over 30 adult patients with solid tumors; (2) intervention: patients were treated with ICIs irrespective of the dosage and duration of the treatment at least one arm; and (3) outcomes: available information regarding SPEN mutation status and OS. SPEN mutation including frameshift, missense, nonsense, splice site, nonstop, and translation start site changes met the pathogenicity criteria. In addition, the references of all trials meeting the above criteria were also examined for possible eligible studies. If the same trial for multiple publications appeared, only the most recent and/or most complete reporting study would be included. The clinical data of cancer patients from five melanoma studies, one lung cancer trial, one renal cancer dataset, and two cohorts, including multiple tumors, was collected. Finally, 2938 patients treated with ICIs were included in this study.

### Statistics

For the difference between tumors and normal tissue, Student’s t test was applied. For the correlation between clinical characteristics and SPEN mutation, statistical significance was determined using the χ2 test, Student’s t test, Mann–Whitney-Wilcoxon rank sum test or Fisher’s exact test. The correlation was evaluated by using Spearman’s ρ correlation coefficient. The survival analysis was analyzed by Kaplan–Meier method and compared using the log-rank test. HR and corresponding 95% CI were calculated by Cox proportional hazards model. *P* < 0.05 was considered statistically significant (*p < 0.05; **p < 0.01; ***p < 0.001; ns, not significant). All statistical analysis was conducted by R 4.2.0 and GraphPad Prism v5.0.

## Results

### Analysis and validation of SPEN expression in pan-cancer

**Fig. 1:**
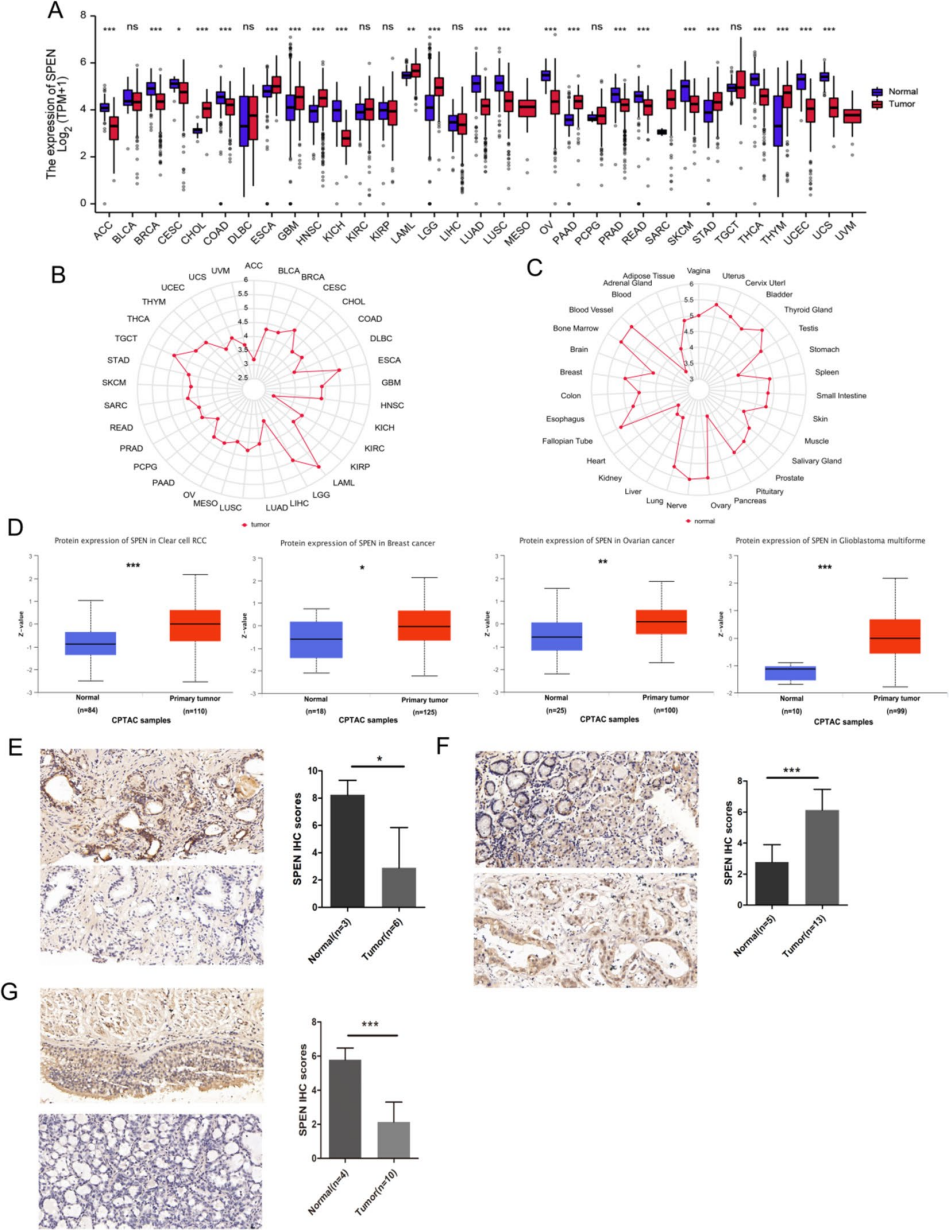
Pan-cancer SPEN expression. **A** SPEN expression between tumor tissues from TCGA database and normal tissues from TCGA and GTEx database. **B**, **C** SPEN expression is shown in tumor and normal tissues from TCGA and GTEx database, respectively. The location of the dot represents the mean value of SPEN expression. **D** We analyze the expression level of the SPEN total protein between normal tissue and primary tissue of clear cell RCC, breast cancer, ovarian cancer, glioblastoma multiforme in the CPTAC dataset. **E**–**G** The protein expression of SPEN in prostate, stomach and lung cancer tissues and corresponding normal tissues was determined by immunohistochemistry (*p < 0.05; **p < 0.01; ***p < 0.001; ns, not significant)

The SPEN expression differences were evaluated by using the TCGA and GTEx datasets. The results revealed that the SPEN expression was significantly upregulated in ESCA, GBM, HNSC, LGG, PAAD, STAD, and THYM, and down-regulated in ACC, BRCA, COAD, LUAD, LUSC, OV, PRAD, READ, SKCM, THCA, UCEC, UCS (Fig. 1A, *p* < 0.001).

Besides, the highest expression of SPEN in LAML, LGG, and ESCA was observed in the TCGA database (Fig. 1B). The highest expression of SPEN in Blood Vessel, Bone Marrow, and Nerve was observed in the GTEx database (Fig. 1C). We also found higher expression of SPEN total protein in the primary tissues of KIRC, BRCA, OV, and GBM (Fig. 1 D, *p* < 0.001) than in normal tissues.

Finally, to further confirm the protein level of SPEN in LUAD, STAD and PRAD patients from the Second Affiliated Hospital of Chongqing Medical University. The representative IHC images of SPEN expression in tumor and normal tissue were shown. Statistical analysis further showed that SPEN expression was significantly upregulated in prostate cancer tissues (Fig. 1E, *p* < 0.05), stomach cancer tissues (Fig. 1F,* p* < 0.001), and lung cancer tissues (Fig. 1G, *p* < 0.001).

### Prognostic analysis of SPEN

We investigated the prognostic significance of SPEN in cancer patients. The results of univariate Cox regression analyses indicated that SPEN expression only notably affected the OS in KIRC (Supplementary Fig. 1A), the DSS in KIRC and LUSC (Supplementary Fig. 1B), the PFS in ACC, KIRC, LIHC, and LUSC (Supplementary Fig. 1C) and the DFS in ACC (Supplementary Fig. 1D). The results of Kaplan–Meier OS indicated that high expression of SPEN was related to the prognosis for the patients with ACC, KIRC and LIHC (Supplementary Fig. 1E-G).

**Fig. 2:**
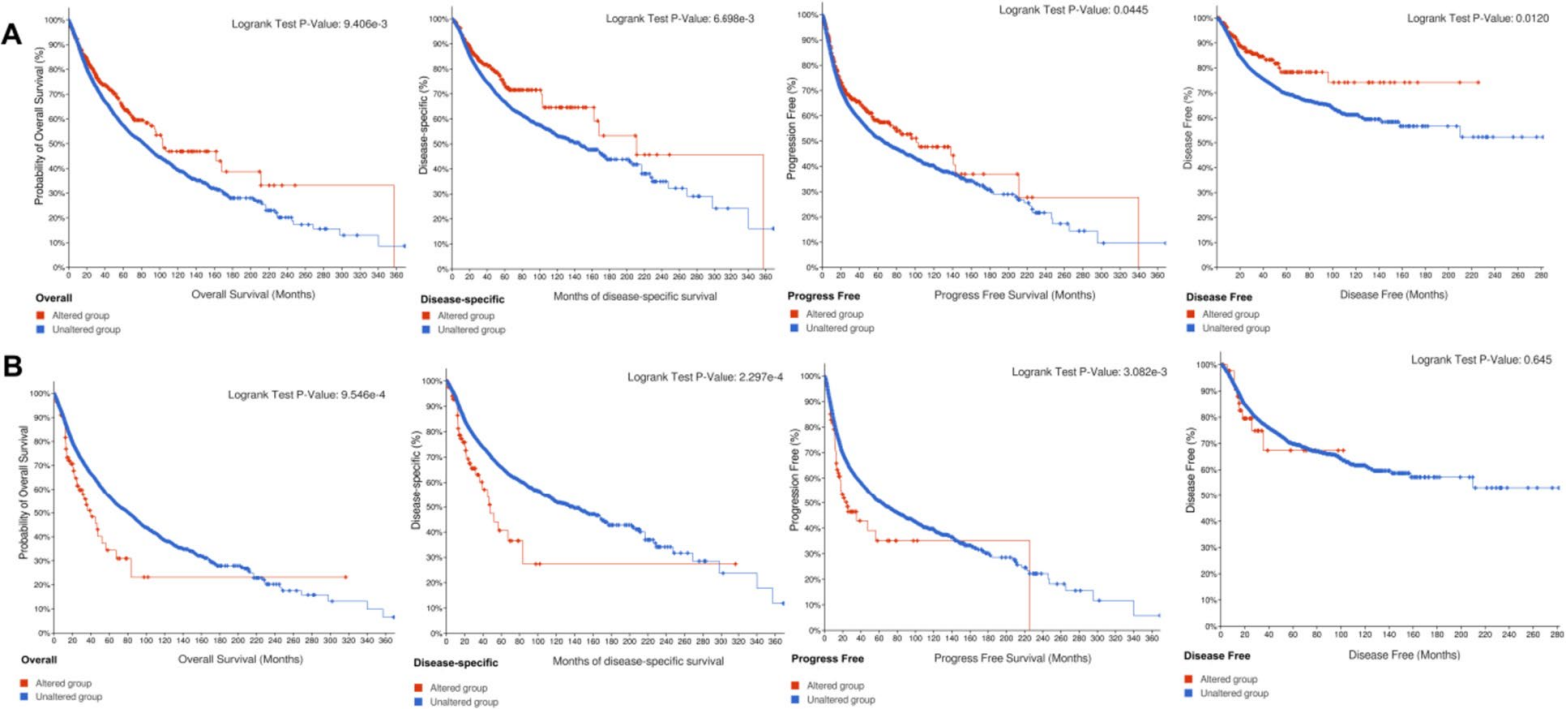
Kaplan–Meier overall survival of SPEN alteration. **A** Overall survival (OS), Disease-specific survival (DSS), Progress-free survival (PFS) and Disease-free survival (DFS) analysis stratified by SETD2 mutation status in pan-cancer from the TCGA cohort. **B** Overall survival (OS), Disease-specific survival (DSS), Progress-free survival (PFS) and Disease-free survival (DFS) analysis stratified by SETD2 CNA status in TCGA pan-cancer

Intriguingly, when we investigated whether the SPEN mutation could translate into cancer prognosis in the TCGA cohort, the results demonstrated that patients with SPEN mutation had better OS (*p* = 0.009), DSS (*p* = 0.007), PFS (*p* = 0.044) and DFS (*p* = 0.012), which implied the prognosis and survival for cancer patients were dependent of SPEN mutant status (Fig. 2A). In addition, copy number alteration (CNA) of SPEN was also associated with poor prognosis in OS (*p* < 0.001), DSS (*p* < 0.001) and PFS (*p* = 0.003), but not DFS (*p* = 0.645), compared with patients without CAN of SPEN in TCGA cohort (Fig. 2B).

### Genetic alteration analysis

**Fig. 3:**
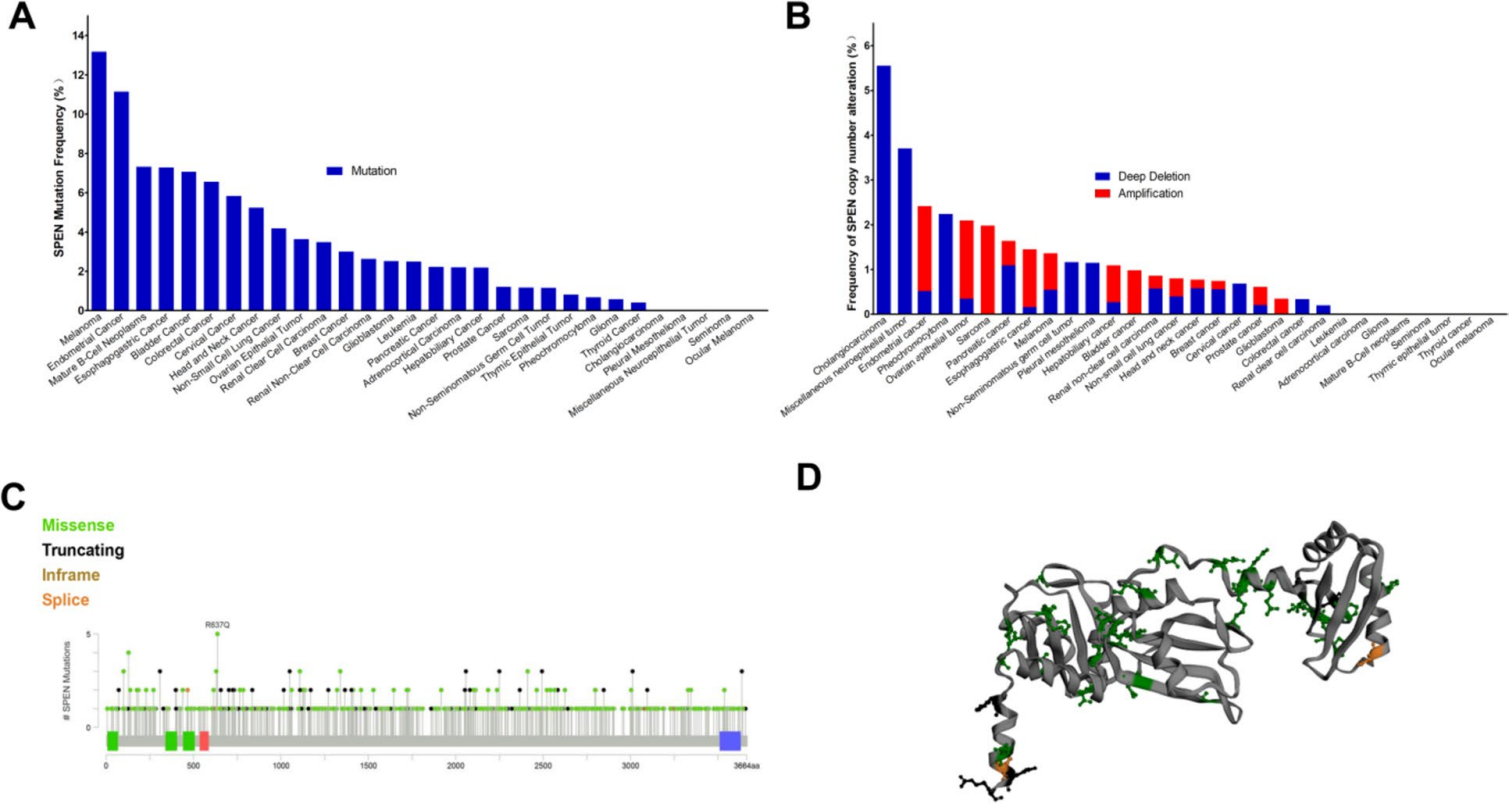
The characteristics of SPEN alteration in TCGA pan-cancer cohort. **A**, **B** The prevalence of SETD2 mutations and CAN across caners. **C** The subtypes and distributions of SPEN alteration. **D** Location of variants on the 3D protein structure of SPEN (Green, mutated amino acid)

The genetic alterations of SPEN were analyzed in the TCGA pan-cancer cohort. Of all 10, 953 patients, 442 (4.00%) harbored SPEN mutations (Fig. 3A) and 75 (0.68%) harbored the copy number alterations (CNA) of SPEN (Fig. 3B). The frequencies of mutation and CNA differed significantly across various tumors. Totally, 569 genetic alterations of SPEN were identified and missense mutation of SPEN is the predominant type, with a frequency of 5.26%. The 5 patients with R637Q mutations were detected. These alterations occurred dispersed manner throughout the whole sequence (Fig. 3C) and D protein structure (Fig. 3D).

### Enrichment analysis of SPEN-related genes

**Fig. 4:**
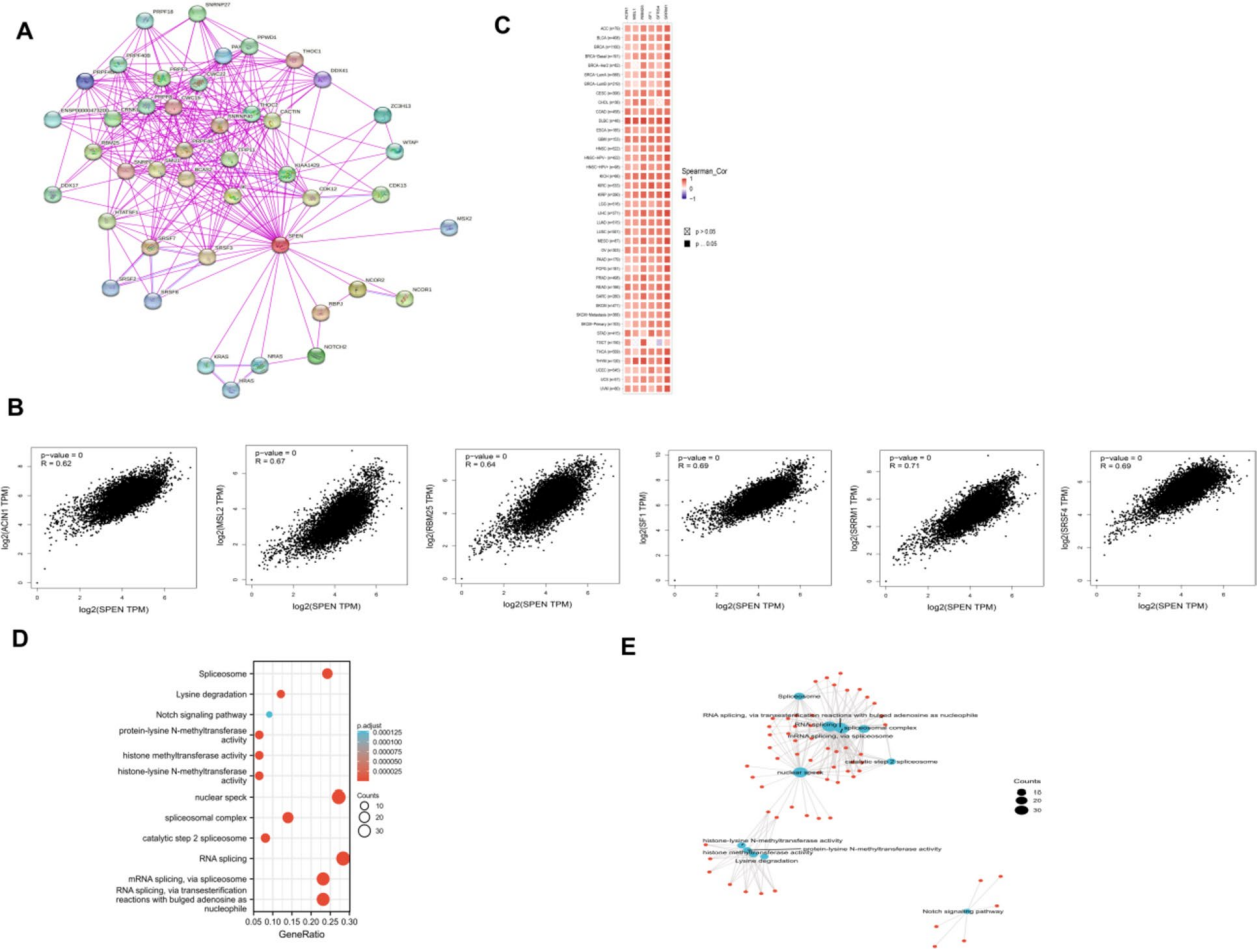
SPEN-related gene enrichment analysis. **A** The experimentally determined SPEN-binding proteins were obtained via the STRING website. **B** The top 100 SPEN-correlated genes were generated using GEPIA2 and the expression correlation between SPEN and selected genes were analyzed, and (**C**) The heatmap presenting the association of SPEN expression and selected genes in pan-cancer. **D**, **E** KEGG pathway and GO analysis based on the SPEN-binding and interacted genes

The interaction network, which included contained 50 SPEN-binding proteins, was created by STRING tool (Fig. 4A). The top 100 genes associated with SPEN expression were obtained via the GEPIA2 tool, and these genes, including apoptotic chromatin condensation inducer 1 (ACIN1) (*r* = 0.62), male-specific lethal 2 (MSL2) (*r* = 0.67), RNA-binding motif protein 25 (RBM25) (*r* = 0.64), sainfoin 1 (SF1) (*r* = 0.69), (serine/arginine-rich splicing factor 4) (*r* = 0.69), (serine/ arginine repetitive matrix 1(SRRM1) (*r* = 0.71), were positively associated with the SPEN (Fig. 4B). A positive association between SPEN and these genes in the majority of detailed cancer type was observed in the heatmap (Fig. 4C). Furthermore, we performed GO and KEGG analysis combined the two datasets to investigate the specific mechanism. The results showed that SPEN and SPEN-binding proteins were significantly involved in Spliceosome and Lysine degradation (Fig. 4D). The relationship of these pathways was demonstrated (Fig. 4E) [28–30].

### Immune infiltration analysis of SPEN

Growing evidence has indicated that tumor-infiltrating immunocytes played a vital role in the survival status of patients [31]. Thus, the relationship of SPEN expression with Stromal, Immune, and ESTIMATE score was investigated in pan-cancers (Supplementary Fig. 2A). Then, the TIMER2.0 database was applied to explore the potential relationship between the SPEN expression and infiltration level of immune cells, including natural killer T cells (NKT), CD4 + T cells, cancer-associated fibroblast (CAF) and regulatory T cells (Tregs) (Supplementary Fig. 2B-E). It was worth noting that SPEN expression was negatively correlated with infiltration level of NKT and T-helper 1 (Th1) cells and positively correlated with CD4 + T cells, cancer-associated fibroblast (CAF) and regulatory T cells (Tregs) in many tumors.

**Fig. 5:**
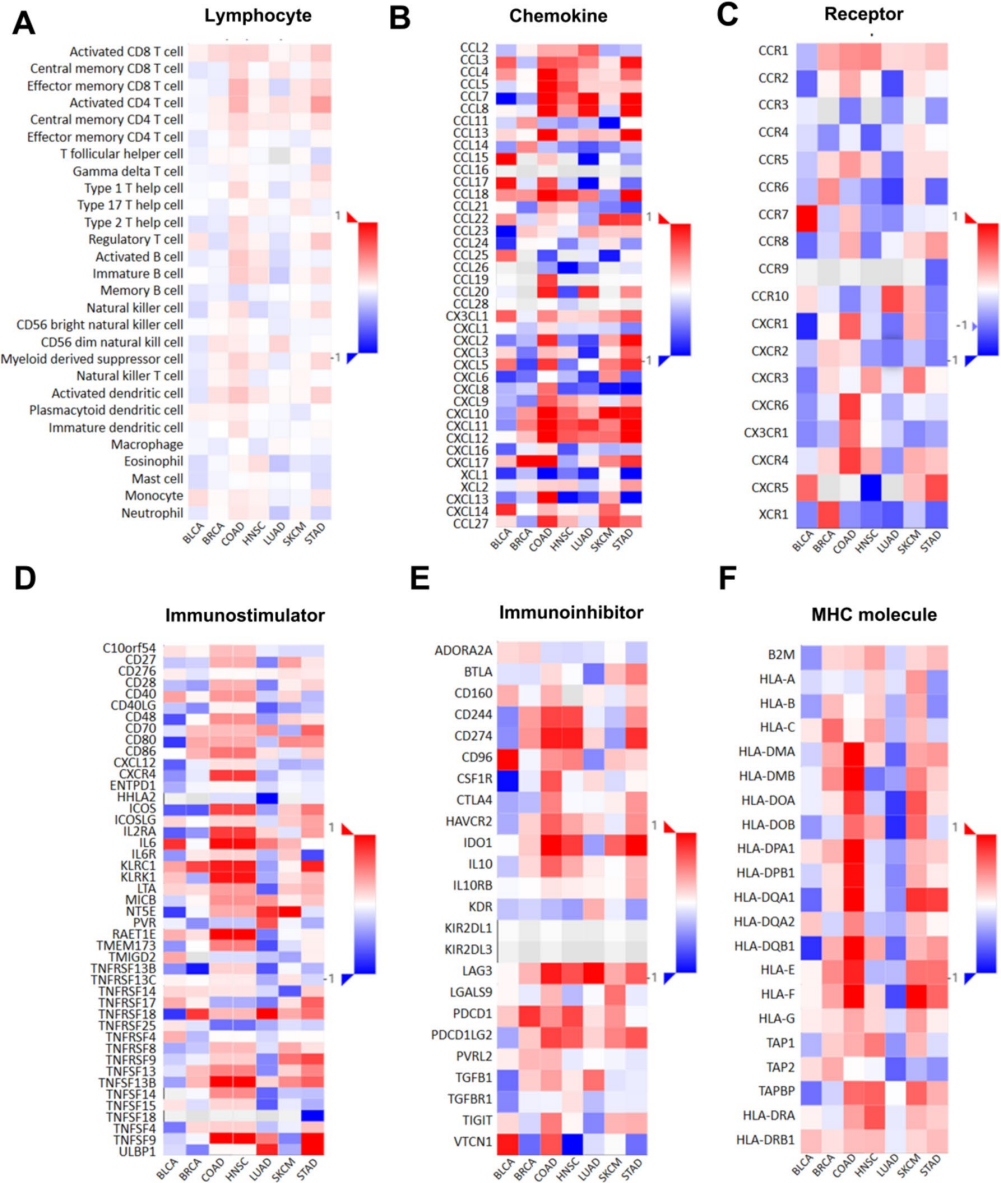
SPEN mutation and immune characters in BLCA, BRCA, COAD, HNSC, LUAD, SKCM, and STAD. **A** The differences of lymphocyte between SPEN mutant patients and SPEN nonmutant patients. **B**-**F** The differences of median gene expression between SPEN mutant patients and SPEN nonmutant patients, including chemokine, receptor, immunostimulators, immunoinhibitors, major histocompatibility complex molecules

Given the important role of SPEN mutation in pan-cancer, the correlation between SPEN mutation and tumor-infiltrating lymphocytes, immunoinhibitors, immunostimulators, major histocompatibility complex molecules (MHC), chemokines, and chemokine receptors was investigated, in STAD (*n* = 35), BRCA (*n* = 32), SKCM (*n* = 30), COAD (*n* = 31), BLCA (*n* = 27), HNSC (*n* = 27) and LUAD (*n* = 20), seven tumors with over 20 SPEN mutant cases in TCGA cohort (Fig. 5A-F). Compared with SPEN nonmutant samples, there were significantly upregulated in SPEN mutant samples. These results inferred that immune response was more active in SPEN mutant cancer and also provided strong evidence that tumor immune phenotype was affected by cancer epigenetic driver mutations.

### Associations of SPEN with immune checkpoints, TMB and MSI

TMB and MSI are recognized as the markers that predict the response to immunotherapy in many cancers. We observed that SPEN expression positively correlated with TMB in ACC, THYM, SARC, and BLCA, while negatively correlated with TMB in BRCA and THCA (Supplementary Fig. 3A; *P* < 0.05). On the contrary, SPEN expression positively correlated with MSI in CESC, SARC, STAD, BLCA, GBM, LUAD, and LUSC, while negatively correlated with MSI in DLBC, SKCM, and THCA (Supplementary Fig. 3B; *P* < 0.05). Then the relationship between SPEN expression and the levels of immune checkpoint gene expression (Supplementary Fig. 3C; *P* < 0.05) indicated that these immune checkpoints are remarkably associated with SPEN expression levels, especially in BLCA, GBM, KIRC, PAAD, PRAD, LUSC, SKCM, and THCA. Furthermore, the relationship between SPEN expression and immunotherapy responses were analyzed using the TIDE algorithm. A regulator prioritization clustering heatmap showed that SPEN expression was related to ICB treatment outcome, T-cell dysfunction/exclusion and phenotypes in CRISPR screens (Supplementary Fig. 4).

**Fig. 6:**
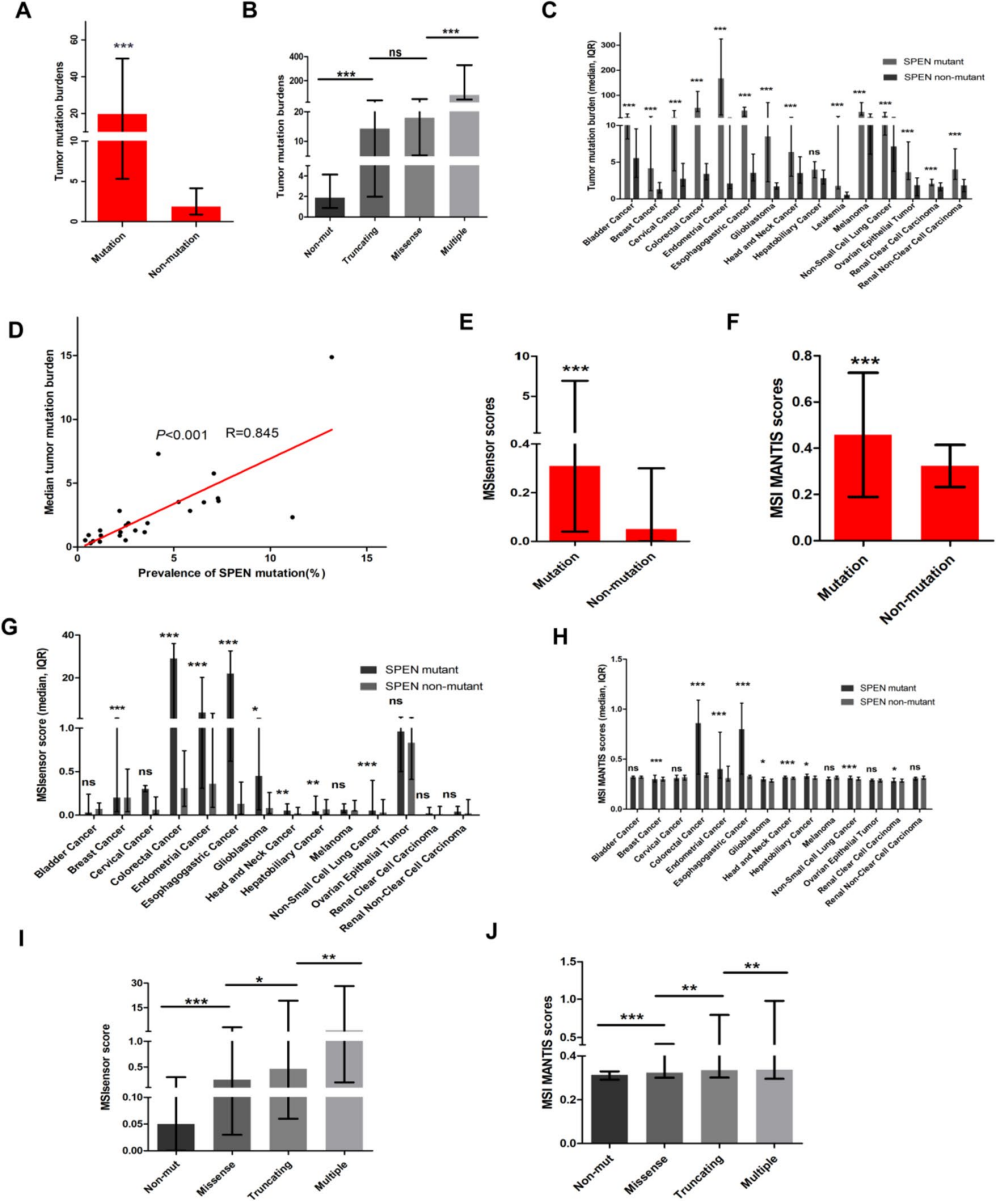
Correlations between SPEN mutation and MSIsensor and MSI MANTIS scores in pan-cancer. **A** TMB in SPEN mutation patients and nonmutation patients. **B** TMB in SPEN nonmutant cancer and different subtypes of SPEN mutant cancer. **C** TMB in various cancer types stratified by SPEN mutation status. **D** The prevalence of SPEN mutation and median TMB in in various cancer types. **E**, **F** MSIsensor and MSI MANTIS scores in SPEN mutation patients and nonmutation patients (**G**, **H**) MSIsensor and MSI MANTIS scores in various cancer types stratified by SPEN mutation status (**I**, **J**) MSIsensor and MSI MANTIS scores were analyzed in SPEN nonmutant cancer and different subtypes of SPEN mutant cancer (*p < 0.05; **p < 0.01; ***p < 0.001; ns, not significant)

Our results implied that SPEN might involve in RNA splicing and processing. To understand the role of SPEN mutation in predicting the response of ICI therapy, we explored the correlation between SPEN mutation and TMB and MSI. The patients with SPEN mutant cancer (median, 19.63; interquartile range, 5.3–49.86) had higher TMB than patients without SPEN mutant cancer (1.9, 0.9–4.1; *P* < 0.0001) in the TCGA cohort (Fig. 6A). Moreover, TMB was significantly different among SPEN missense mutant cancer (18.0, 5.3–45.7), and cancer with multiple mutations (79.0, 42.1–329.0; Fig. 6B), and TMB stratified by SPEN mutation status in different cancer type were shown in Fig. 6C. A remarkable relationship between the frequencies of SPEN mutation and median TMB was observed in different cancer types (*R* = 0.845; *P* < 0.001; Fig. 6D). Then, MSIsensor and MSI MANTIS scores were used to estimate the MSI status of SPEN mutation. MSIsensor and MSI MANTIS scores in patients with SPEN mutant cancer were significantly higher than the patient with SPEN non-mutant cancer (*P* < 0.0001; Fig. 6E, F). There was no correlation between the frequency of SPEN mutation and median MSIsensor and MSI MANTIS scores (R = 0.38; *P* = 0.07; R = -0.032; *P* = 0.88, Supplementary Table 1). The associations between MSIsensor and MSI MANTIS scores and SPEN mutation in different cancer types were shown in Fig. 6G, H. These scores showed significant differences among various subtypes of SPEN mutation (Fig. 6I, J). However, there was no association between TMB, MSIsensor, and MSI MANTIS scores and the CAN of SPEN (Supplementary Fig. 5).

### Association of SPEN with DNA mismatch repair (MMR)

**Fig. 7:**
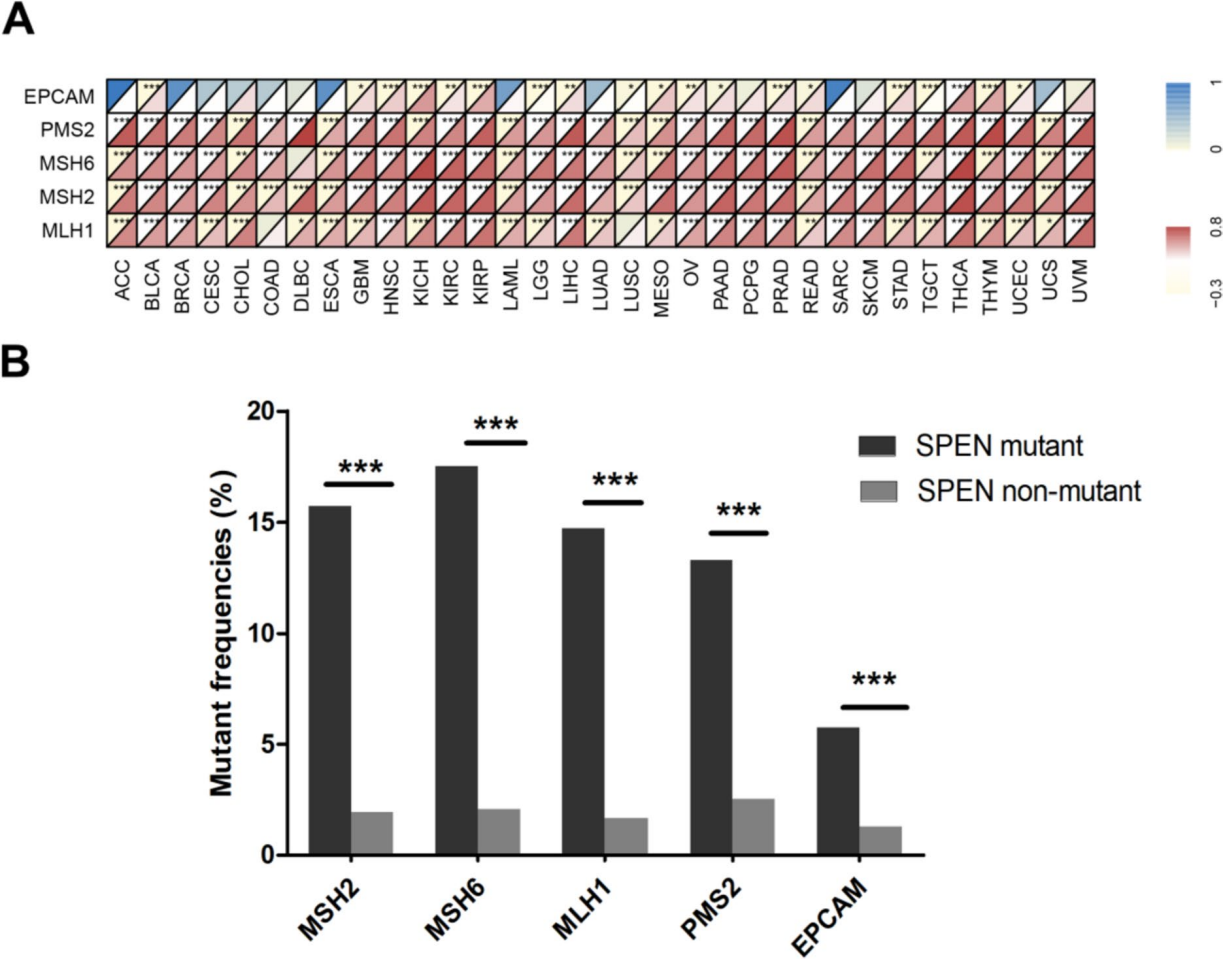
Correlations between SPEN and five MMR genes in pan-cancer. **A** Spearman’s correlation analysis of SPEN expression with expression levels of five MMR genes in various cancer types. **B** The mutant frequencies of five MMR genes in SPEN mutant and nonmutant cancer (*p < 0.05; **p < 0.01; ***p < 0.001)

The MMR pathway, which mainly consisted of MSH2, MSH6, PMS2, MLH1, and EPCAM, played a pivotal role in maintaining DNA replication fidelity and genome stability, which is related to the molecular character of MSI and predisposed to cancer. Thus, the potential relationship between SPEN and MMR needs to be investigated in pan-cancer (Fig. 7). The results suggested that SPEN expression was significantly associated with MMR genes in almost all cancer. Of note, compared with patients without SPEN mutant cancer, patients with SPEN mutant cancer harbored more MMR mutant genes (MSH2, 1.97% vs.15.73%; MSH6, 2.08% vs.17.52%; MLH1, 1.67% vs.14.73%; PMS2, 2.55% vs.13.30%; EPCAM, 1.30% vs.5.75% *P* < 0.0001 for all five genes).

### Association of SPEN mutation with immunotherapy

**Fig. 8:**
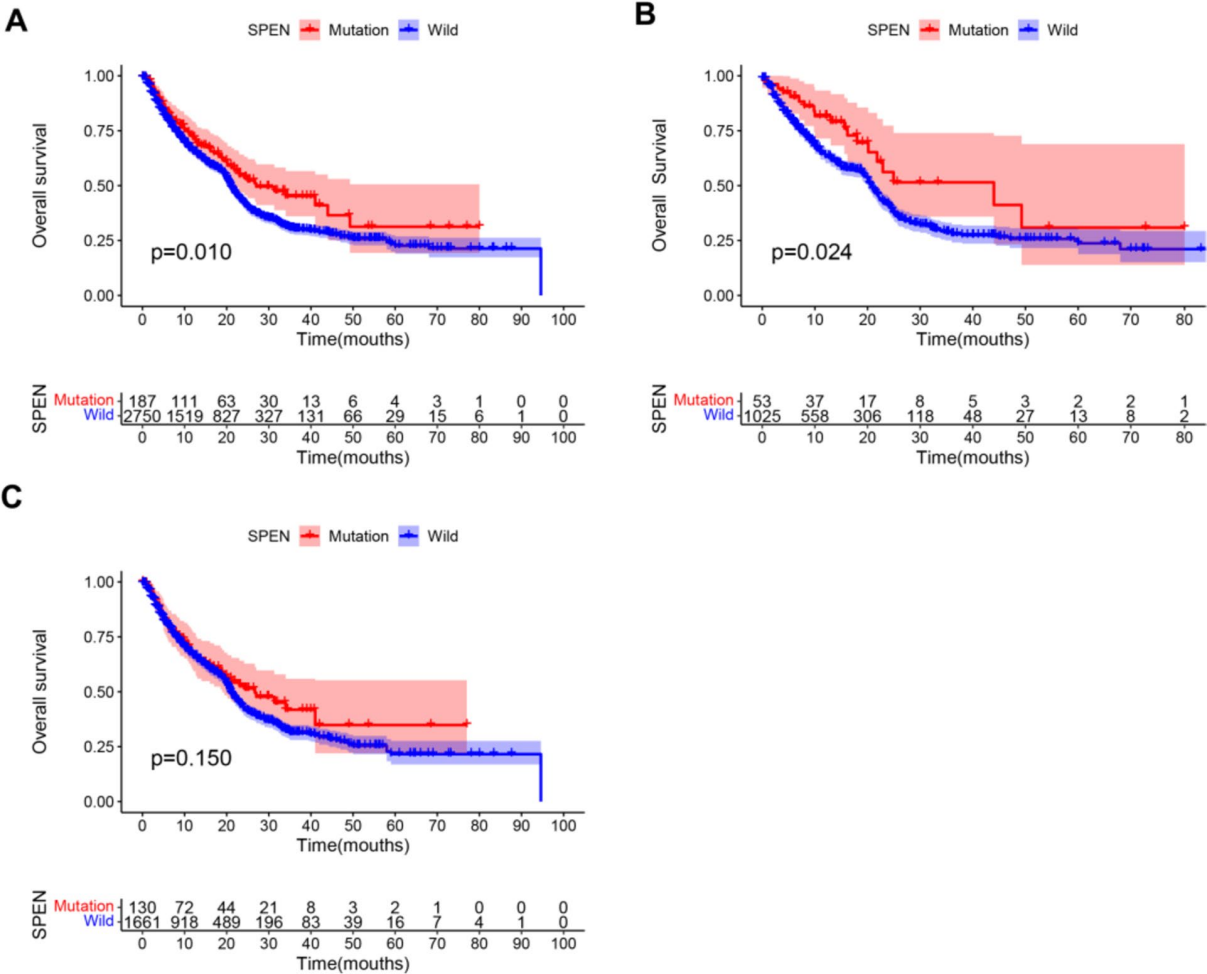
SPEN mutation and the clinical outcome of immunotherapy. **A** Kaplan–Meier survival analysis stratified by SPEN mutation status in 2938 cancer patients treated with immune checkpoint inhibitors. **B**, **C** Kaplan–Meier survival analysis stratified by SPEN mutation status in female and male cancer patients treated with immune checkpoint inhibitors

To evaluate whether SPEN mutation is a predictive biomarker for cancer immunotherapy, we analyze the data, including 2,938 patients from 9 studies that receive ICIs treatment (Supplement Table 2) [32–40]. The results showed that SPEN mutation was significantly associated with better OS (HR, 0.74; 95%CI, 0.59–0.93 *P* = 0.01; Fig. 8A), and this association remained existed in female patients (HR, 0.60; 95%CI, 0.38–0.94 *P* = 0.024; Fig. 8B), but not in male patients (HR, 0.82; 95%CI, 0.62–1.08 *P* = 0.150; Fig. 8C). Finally, we specifically analyze the association between SPEN mutation and OS using univariable and multivariable Cox analysis. As shown in Table 1, SPEN mutation was still a predictive biomarker in patients treated with ICIs, especially in women (Table 2). The above data indicated that SPEN mutation significantly predicted the efficacy of immunotherapy.

**Table 1:** Cox analysis of the association between SPEN mutation and overall survival

Variables	Univariable analysis			Multivariable analysis		
	HR	95% CI	*P* value	HR	95% CI	*P* value
Age (> 60 vs ≤ 60)	0.94	0.84–1.05	0.296	0.95	0.84–1.07	0.351
Gender (male vs female)	0.94	0.84–1.04	0.244	0.92	0.81–1.04	0.166
Cancer type						
Melanoma	1.00	Reference		1.00	Reference	
Lung cancer	1.37	(1.20–1.57)	< 0.001	1.38	(1.17–1.63)	< 0.001
Other cancer	1.50	(1.32–1.70)	< 0.001	1.43	(1.22–1.67)	< 0.001
Treatment (combo vs mono)	0.61	0.50–0.75	< 0.001	0.59	(0.48–0.73)	< 0.001
TMB (> median vs ≤ median)	0.73	0.65–0.82	< 0.001	0.79	(0.70–0.90)	< 0.001
SPEN (Mutation vs Wild)	0.74	0.59–0.93	0.010	0.70	(0.51–0.95)	0.023

**Table 2:** Cox analysis of the association between SPEN mutation and overall survival in female patients

Variables	Univariable analysis			Multivariable analysis		
	HR	95% CI	*P* value	HR	95% CI	*P* value
Age (> 60 vs ≤ 60)	0.80	0.67–0.94	0.009	0.80	0.66–0.97	0.021
Cancer type						
Melanoma	1.00	Reference		1.00	Reference	
Lung cancer	1.43	(1.15–1.77)	0.001	1.42	(1.08–1.85)	0.01
Other cancer	1.85	(1.49–2.31)	< 0.001	1.61	(1.24–2.09)	< 0.001
Treatment (combo vs mono)	0.76	0.56–1.03	0.080	0.70	(0.50–0.97)	0.034
TMB (> median vs ≤ median)	0.73	0.60–0.88	0.001	0.82	(0.68–1.00)	0.054
SPEN (Mutation vs Wild)	0.60	0.38–0.94	0.024	0.50	(0.27–0.91)	0.023

## Discussion

Immune evasion is pivotal role in accelerating tumor growth and metastasis mainly exploiting tumor surface antigen modulation and tumor-induced immunosuppression to achieve this process [41, 42]. However, the emergence of immunotherapy, targeting PD-1/ PD-L1 and CTLA-4 pathway blockades to reverse immunosuppressive, appears to become a successful anticancer strategy and is able to induce long-term tumor remission in patients with advanced malignant tumors [43]. The limitation of immunotherapy is a low response rate, and identifying predictive biomarkers to screen patients who respond to immunotherapy and preciously monitoring its efficacy is of great clinical significance [44–46].

Aberrant X chromosome inactivation (XCI) will result in the silencing of X-linked genes at the local and chromosomal levels, which might alter the expression of cancer-related and cause the development of tumors [47, 48]. SPEN is essential for XCI, which excludes polymerase from DNA to prevent gene expression [49]. In this study, we investigated the role of SPRN in tumorigenesis and whether it could serve as a biomarker for predicting immunotherapy.

Our results suggested that abnormal SPEN expression related to prognostic values in some cancer types. SPEN expression closely related to the levels of immune infiltration. Moreover, SPEN expression significantly correlated with Immune Checkpoints, TMB, MSI and MMR in various cancers. Intriguingly, we found that SPEN mutation indicated better prognostic values, and also served as a strong prognostic factor for cancer patients treated with ICI therapy. Cancer patients with SPEN mutation had distinct tumor immune signatures, higher TMB and MSI and more MMR mutant genes, and further explain an underlying mechanism of the predictive value of SPEN mutation on the immunotherapy efficacy.

An increasing number of studies have been performed to identify biomarkers of the response to ICI therapies. Molecular analysis of tumors has implied that somatic nonsynonymous coding mutations correlated with tumor immunogenicity and response to immune checkpoint therapy [33, 50]. TMB, which could evaluate tumor immunogenicity, is a potential emerging biomarker for associating with response to immunotherapy using ICIs. However, not all patients with a high TMB were associated with potential clinical benefits, which lead to uncertainty in response to ICIs [51, 52]. Tumors with mismatch repair deficiency (MMR-d) and high microsatellite instability (MSI-H) had been validated for rendering the tumors immunogenic and increasing response to ICI therapy, but imperfect predictive biomarkers in most cancers with controversial results across different trials [53, 54]. Recently, some evidence indicates that some specific genetic mutations played an essential role in tumors' immunogenic and infiltration levels of immune cells, leading to distinct immune responses [55]. Therefore, we focused on identifying a novel biomarker to better evaluate the efficacy of ICIs treatment. First, we found that SPEN expression was correlated with TMB and MSI in ACC, THYM, SARC, and BLCA. Especially, SPEN mutation significantly correlated with TMB and MSI in pan-caner. In addition, SPEN expression significantly affected MMR gene expression in almost all cancer, and patients with SPEN mutant cancer harbored more MMR mutant genes. Second, after SPEN mutation predicting the clinical outcomes of immunotherapy was established, we further used clinical data, including 2,938 patients from 9 studies to validate that SPEN mutation could serve as a good biomarker. Besides, SPEN mutation was significantly associated with OS in patients treated with ICI therapy in pan-caner. Third, the multivariate Cox regression analysis was performed, and we found that SPEN mutation was independent of cancer type in predicting prognosis. These results supported that SPEN mutation was a potential biomarker for cancer patients with ICIs treatment.

The diversity of tumor-infiltrating lymphocytes was crucial for response to immunotherapy. The infiltration level of immune cells was associated with anti–PD-1/PD-L1 therapy [56]. Our results suggested that SPEN expression is positively associated with ESTIMATEScore in most cancer types, indicating the high purity of the tumor and better prognosis in cancer. Immune cell infiltration analysis showed a negative association between NKT and Th1 cells and SPEN expression, while CAF and Tregs cells were positively correlated with SPEN expression. Furthermore, SPEN mutation was significantly correlated with tumor-infiltrating lymphocytes, immunoinhibitors, immunostimulators, MHC, chemokines, and chemokine receptors, and SPEN expression was positively correlated with immune checkpoint genes. These results further explained the underlying mechanism that SPEN could affect the efficacy of immunotherapy.

Numerous studies have shown that the incidence of many cancers had distinguished sex difference, with men having a higher incidence and mortality rate of malignancies than women [57, 58]. Abnormal inactivation of the X chromosome may be an essential factor leading to this sex difference [59]. SPEN is essential for XCI, and our results showed that women had a better prognosis compared with men who received ICIs treatment. In addition, SPEN mutation was an independent biomarker after adjusting for confounding factors, including age, cancer type, treatment strategy, and TMB.

Several limitations should be considered. First, the heterogeneity of the included study needed to be further evaluated. Second, patients in this study were treated with ICIs from different pharmaceutical companies, which might lead to drug bias. Third, insufficient patients with each cancer type may restrict our analysis for different cancer types. In the future, a prospective study with a larger sample size of cancer patients treated with ICI is warranted to explore the predictive value of SPEN mutation.

## Conclusion

In conclusion, our study determined the expression of SPEN was significantly different in various cancers from the TCGA cohort and clinical tumor samples. Besides, our results showed that SPEN mutation has distinct tumor immune signatures and correlates with higher TMB and MSI. Furthermore, SPEN mutation is a biomarker in predicting prognosis and clinical benefit of ICIs treatment and needs to be validated in a prospective study.

### Supplementary Information

**Additional file 1.**

**Additional file 2:** **Supplement Table 1.** The frequency of SPEN mutation and median TMB, MSIsensor and MSI MANTIS scores. **Supplement Table 2.** Characteristics of studies included in this study.

**Additional file 3**.

## Data Availability

The original data presented in the study are included in the article/supplementary materials. Further inquiries can be directed to the corresponding authors.
