# Supplementary material for: Pan-cancer analysis identifies SPEN mutation as a predictive biomarker with the efficacy of immunotherapy
Source: BMC Cancer. 2023 Aug 24;23:793. doi: 10.1186/s12885-023-11235-0 (PMC10463702; doi:10.1186/s12885-023-11235-0)
Supplement: Supplementary file 1 — Additional file 1. [file 12885_2023_11235_MOESM1_ESM.docx]

Supplement Figure 1: Prognostic Analysis of SPEN expression. (A-D) Forest map shows the univariate cox regression results of SPEN for OS, DSS, PFS and DFS in TCGA pan-cancer. (E-G) Kaplan-Meier overall survival of SPEN is shown in ACC, KIRC and LIHC from TCGA database.

Supplement Figure 2: Associations of SPEN expression with immune infiltration. (A) The heatmap displaying the correlations of SPEN expression with ImmuneScore, StromalScore, and ESTIMATEScore in pan-cancer. (B-E) The correlation between SPEN and infiltration level of nature killer T cells (NKT), CD4+ T cells, cancer-associated fibroblast (CAF) and regulatory T cells (Tregs) using TIMER2 database.

Supplement Figure 3: Correlations between SPEN expression and TMB, MSI and immune checkpoints in pan-cancer. (A) Radar map of correlation between SPEN expression and TMB. The red reveals the correlation coefficient. (B) Radar map of correlation between SPEN expression and MSI. The blue reveals the correlation coefficient. (C) Correlation between SPEN expression and immune checkpoints. (*p < 0.05; **p < 0.01; ***p < 0.001 and ****p < 0.0001.)

Supplement Figure 4: The regulator prioritization clustering heatmap showed the association between SPEN expression and immunotherapy responses.

Supplement Figure 5: Correlations between SPEN copy number alteration (CNA) and TMB, MSIsensor and MSI MANTIS scores in pan-cancer. (A) TMB in SPEN CNA samples and non-CNA samples. (B) MSI MANTIS scores in SPEN CNA samples and non-CNA samples. (C) MSIsensor scores in SPEN CNA samples and non-CNA samples.

**SPEN Expression and clinical Extraction**

The SPEN expression of The Cancer Genome Atlas (TCGA) and corresponding normal samples data of the Genotype-tissue expression (GTEx) database were extracted from from the UCSC Xena database (<https://xenabrowser.net/datapages/>) by using R4.2.0 software. Supplementary file showed that detailed the information of code files and corresponding SPEN expression data were described in Supplementary file (“Exoression. R” and “SPEN Exp.xlsx”)

**Survival analysis of SPEN expression**

The survival data of pan-cancer was downloaded from UCSC Xena database (<https://xenabrowser.net/datapages/>) and the overall survival of SPEN was extracted by using R4.2.0 software. Supplementary file showed that detailed the information of code files, including Kaplan-Meier survival and cox regression (OS, DSS, PFS and DFS), and corresponding overall survival data were described in Supplementary file (“Kaplan-Meier survival”, “Survival time.R”, “OS.R”, “DSS.R”, “PFS.R”, “DFS.R” ,“SPEN expression overall survival data.xlsx”)

**Survival analysis of SPEN mutation**

9 studies involved 2,938 patients that receive ICIs treatment were included [[1-9](#_ENREF_1)] (Supplement Table2). Supplementary file showed that detailed the information of code files and corresponding survival data (overall, female and male) were described in Supplementary file (“SPEN mutation survival.R” and “SPEN mutation survival data.xlsx”)

**Correlation between SPEN expression and TMB, MSI.**

The data of TMB and MSI were downloaded from TCGA database. The correlation between SPEN expression and TMB and MSI was analyzed by using R4.2.0 software. Supplementary file showed that detailed the information of code files, including TMB, MSI, and corresponding TMB and MSI data were described in Supplementary file (“TMB”, “TMB rader.R”, “MSI”, “MSI rader.R”, “TMB data.xlsx” and “MSI data.xlsx”)

**Correlation between SPEN expression and MMR and immune checkpoints**

The data of Correlation between SPEN expression and MMR and immune checkpoints was obtained from the TIMER2 database (http:// timer.cistrome.org/). The heatmap was obtained by using using R4.2.0 software and corresponding information of code files in the supplementary file (“heatmap.R”).

**Reference**

1. Miao D, Margolis CA: Genomic correlates of response to immune checkpoint therapies in clear cell renal cell carcinoma. 2018, 359(6377):801-806

2. Miao D, Margolis CA: Genomic correlates of response to immune checkpoint blockade in microsatellite-stable solid tumors. 2018, 50(9):1271-1281.

3. Colognori D, Sunwoo H, Kriz AJ, Wang CY, Lee JT: Xist Deletional Analysis Reveals an Interdependency between Xist RNA and Polycomb Complexes for Spreading along the Inactive X. Mol Cell 2019, 74(1):101-117.e110.

4. Hugo W, Zaretsky JM, Sun L, Song C, Moreno BH, Hu-Lieskovan S, Berent-Maoz B, Pang J, Chmielowski B, Cherry G et al: Genomic and Transcriptomic Features of Response to Anti-PD-1 Therapy in Metastatic Melanoma. Cell 2016, 165(1):35-44.

5. Liu D, Schilling B: Integrative molecular and clinical modeling of clinical outcomes to PD1 blockade in patients with metastatic melanoma. 2019, 25(12):1916-1927.

6. Riaz N, Havel JJ, Makarov V, Desrichard A, Urba WJ, Sims JS, Hodi FS, Martín-Algarra S, Mandal R, Sharfman WH et al: Tumor and Microenvironment Evolution during Immunotherapy with Nivolumab. Cell 2017, 171(4):934-949.e916.

7. Samstein RM, Lee CH, Shoushtari AN: Tumor mutational load predicts survival after immunotherapy across multiple cancer types. 2019, 51(2):202-206.

8. Snyder A, Makarov V, Merghoub T, Yuan J, Zaretsky JM, Desrichard A, Walsh LA, Postow MA, Wong P, Ho TS et al: Genetic basis for clinical response to CTLA-4 blockade in melanoma. N Engl J Med 2014, 371(23):2189-2199.

9. Van Allen EM, Miao D, Schilling B, Shukla SA, Blank C, Zimmer L, Sucker A, Hillen U, Foppen MHG, Goldinger SM et al: Genomic correlates of response to CTLA-4 blockade in metastatic melanoma. Science 2015, 350(6257):207-211.


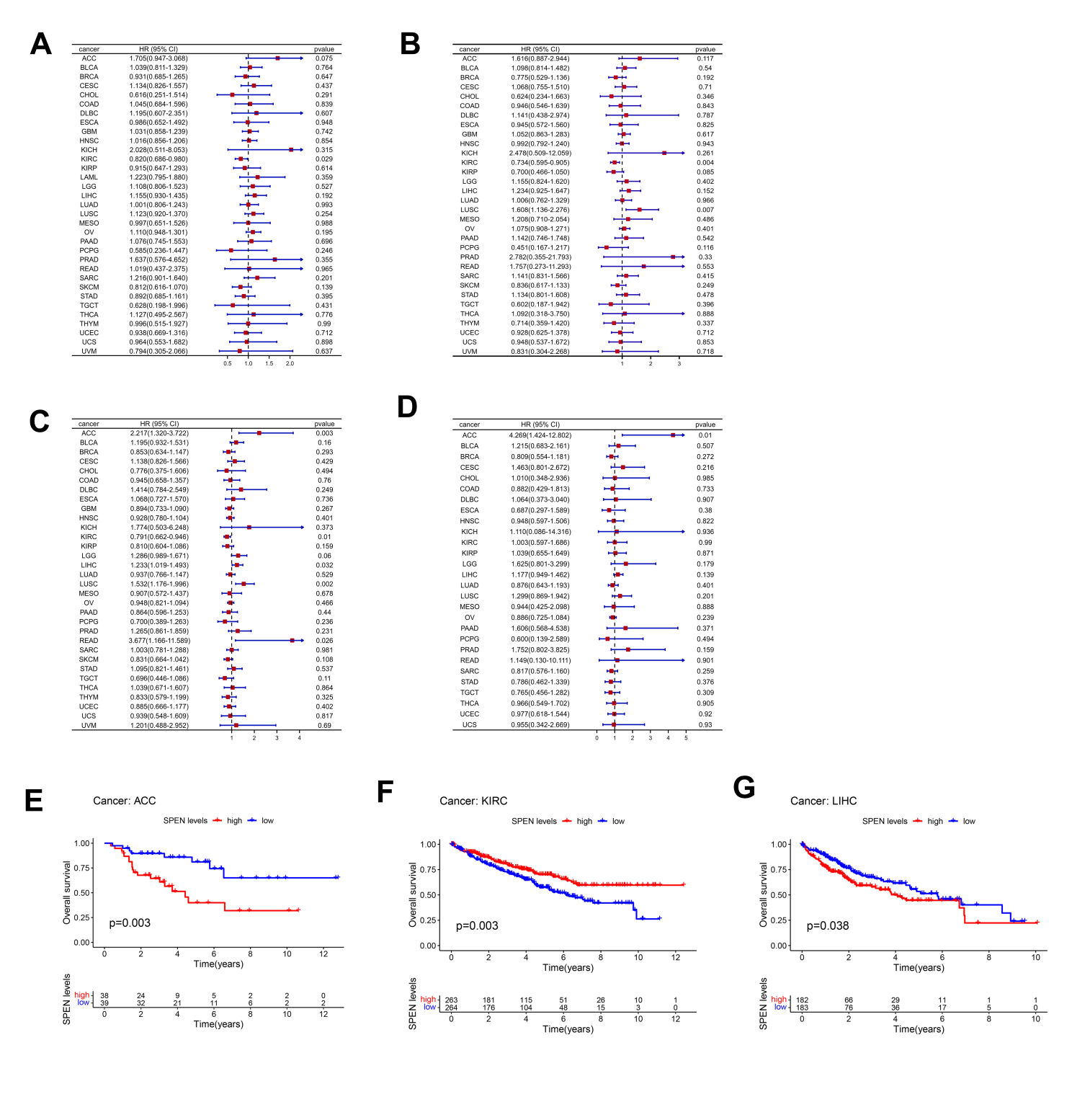


Supplement Figure 1


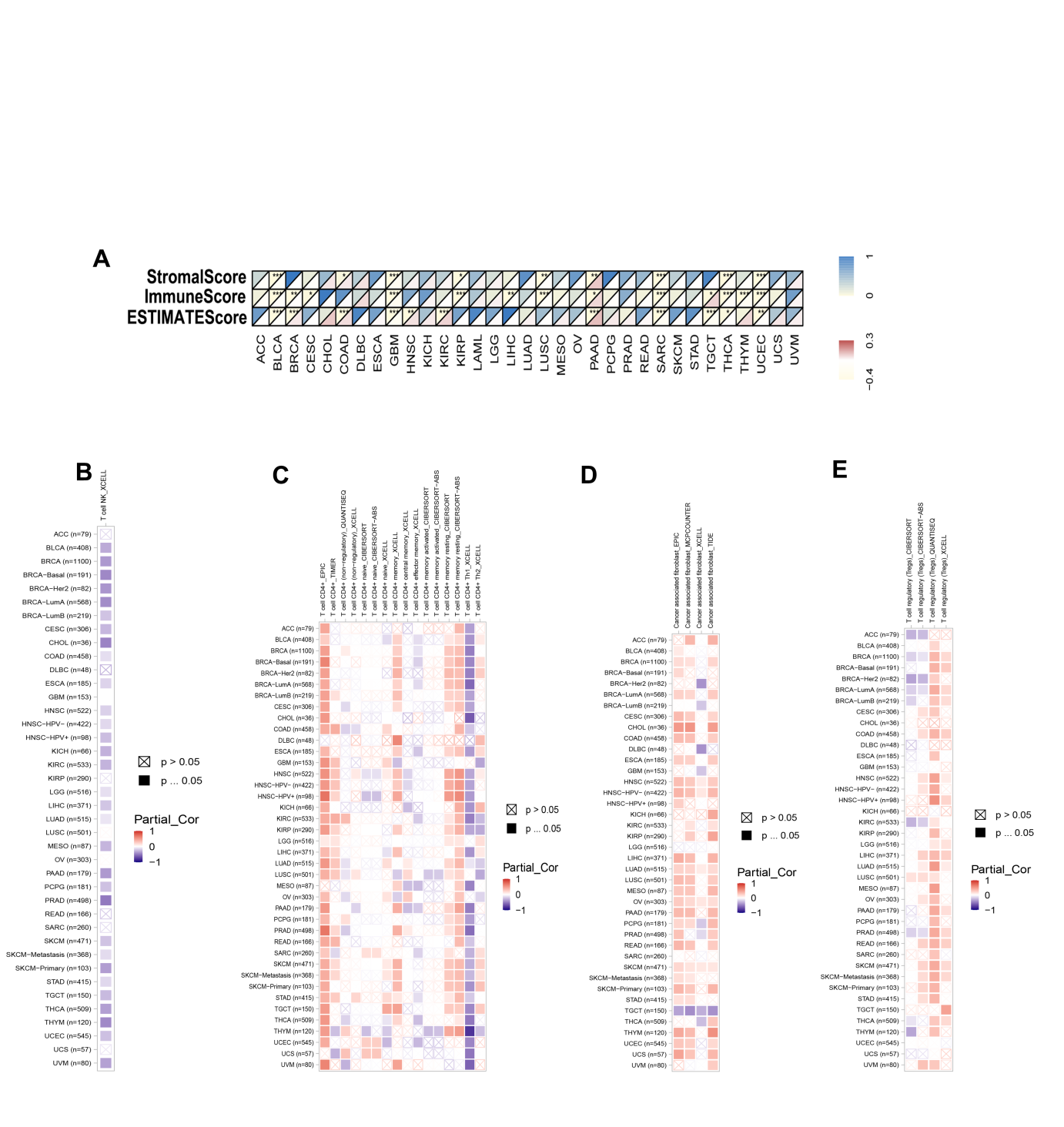


Supplement Figure 2


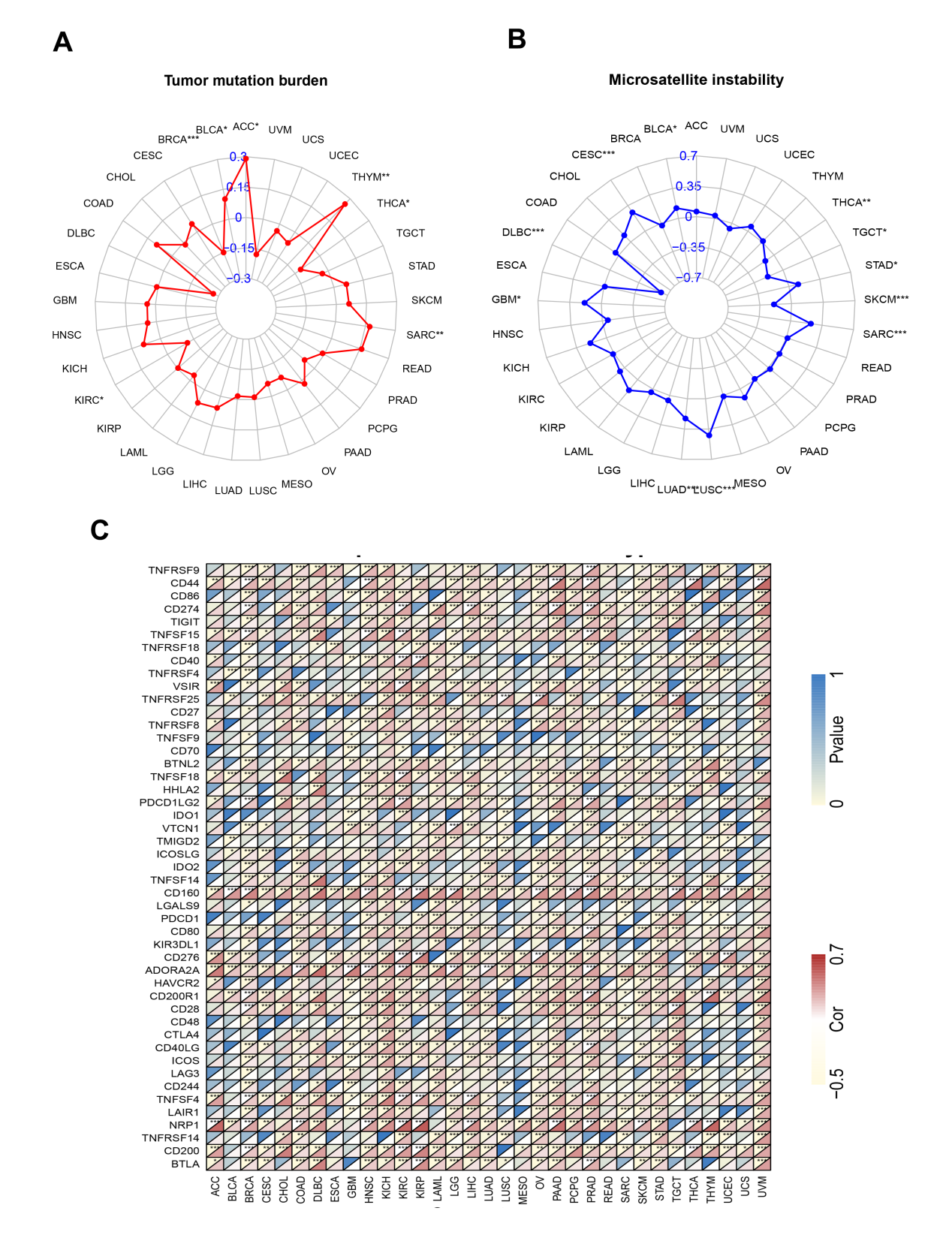


Supplement Figure 3
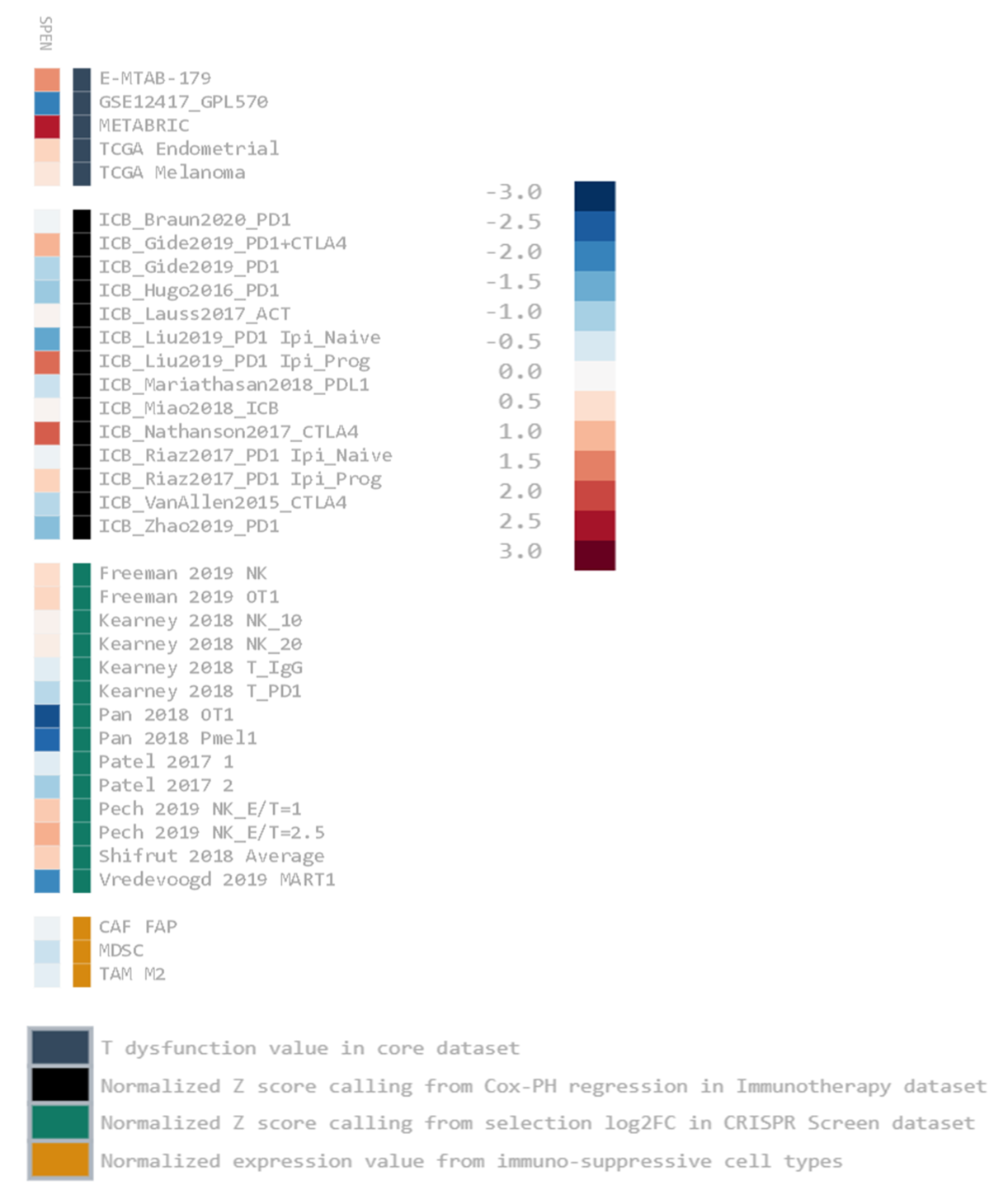


Supplement Figure 4


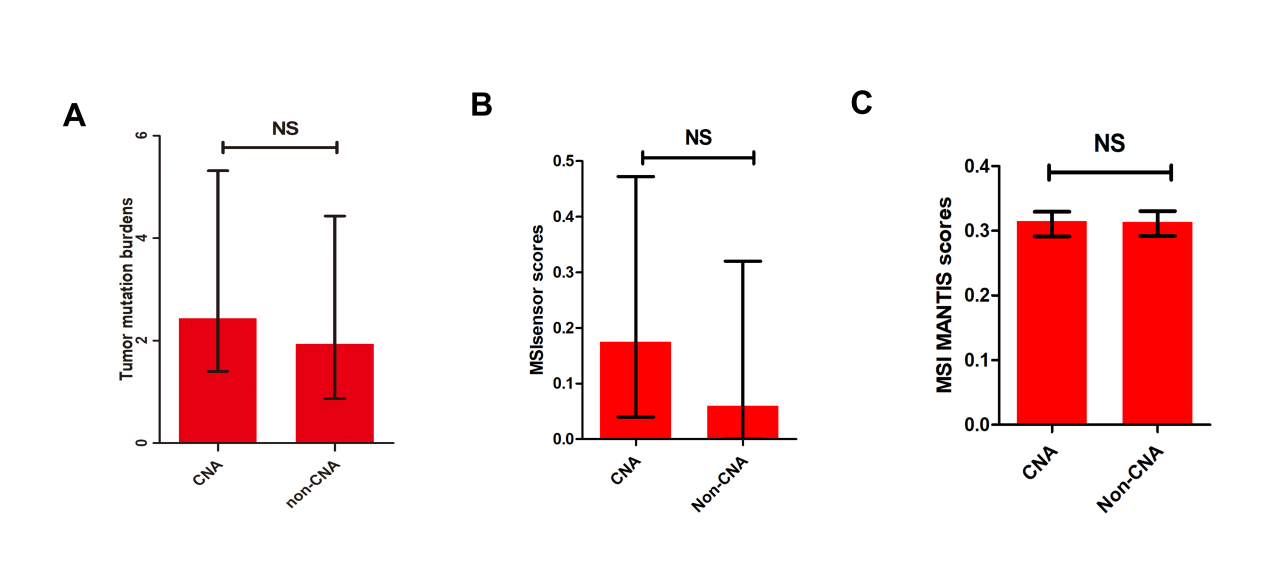


Supplement Figure 5
