# Supplementary material for: Pan-cancer analysis identifies SPEN mutation as a predictive biomarker with the efficacy of immunotherapy
Source: BMC Cancer. 2023 Aug 24;23:793. doi: 10.1186/s12885-023-11235-0 (PMC10463702; doi:10.1186/s12885-023-11235-0)
Supplement: Supplementary file 3 — Additional file 3. [file 12885_2023_11235_MOESM3_ESM.pdf]

KEGG Database Project
Kanehisa Laboratories
www.kanehisa.jp

Ref: 230534

Permission is granted to BMC Cancer to publish under the CC BY 4.0 open access license the following KEGG pathway map images in the article "Pan-cancer analysis identifies SPEN mutation as a predictive biomarker with the efficacy of immunotherapy " written by Chuan Liu and colleagues:

- Spliceosome - Homo sapiens (human) (hsa03040)
- Lysine degradation - Homo sapiens (human) (hsa00310)
- Notch signaling pathway - Homo sapiens (human) (hsa04330)
- Ubiquitin mediated proteolysis - Homo sapiens (human) (hsa04120)
- Thyroid hormone signaling pathway - Homo sapiens (human) (hsa04919)
- Nucleocytoplasmic transport - Homo sapiens (human) (hsa03013)

subject to the condition that the original source is acknowledged by citing at least one KEGG paper.

Permission granted: Naomi Takahashi

Naomi Takahashi, Kanehisa Laboratories

Date: 4 April 2023

Copyright holder: Kanehisa Laboratories
